# Supplementary material for: Exonuclease-enhanced prime editors
Source: Nat Methods. 2024 Feb 1;21(3):455–64. doi: 10.1038/s41592-023-02162-w (PMC10927552; doi:10.1038/s41592-023-02162-w)
Supplement: Supplementary file 1 — Supplementary Figs. 1 and 2, FACS gating strategy. [file 41592_2023_2162_MOESM1_ESM.pdf]

nature methods

Article https://doi.org/10.1038/s41592-023-02162-w

Exonuclease-enhanced prime editors

In the format provided by the authors and unedited

Table of Contents

Supplementary Figure 1 | Additional comparison of 'Exo-PE' and 'PE2' performance on 11 loci. – page 2

Supplementary Figure 2 | Reproducibility of editing results. – page 3

FACS gating strategy – pages 4-6

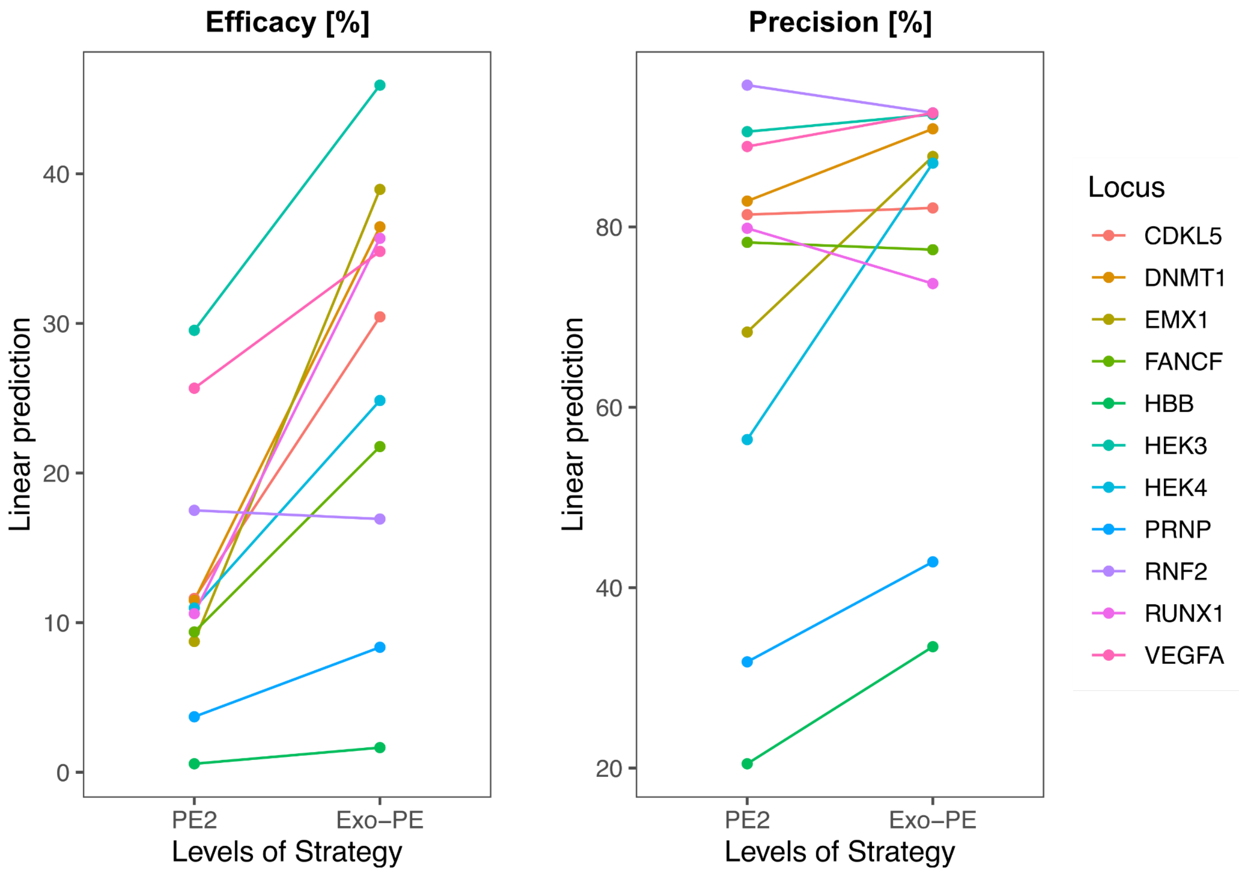

Supplementary Figure 1 | Additional comparison of 'Exo-PE' and 'PE2' performance on 11 loci. Efficacy and precision data were combined for all loci on which direct comparisons of 'PE2' and 'Exo-PE' were conducted (data taken from Figures 4c, 5, 6, Extended Data Figure 7a). The predicted editing efficacy (left) and precision (right) from the linear model (~ Locus * Strategy * iPE-N/iPE-C) averaged over iPE-N/C are plotted color-coded for all 11 loci using the emmeans package in R (3.4.1).

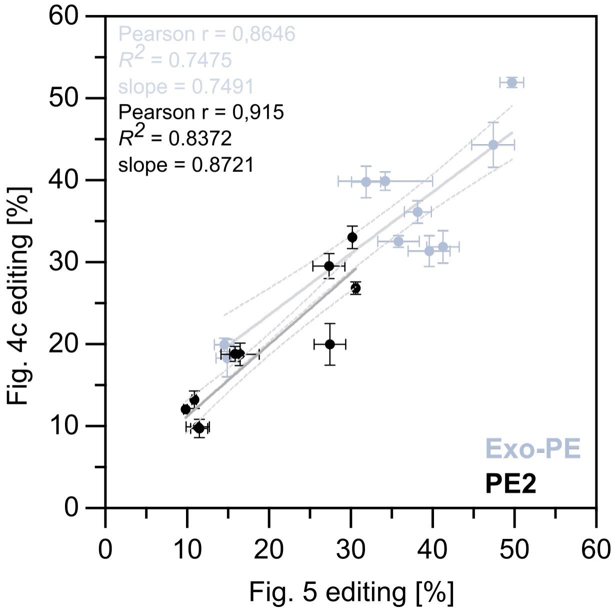

Supplementary Figure 2 | Reproducibility of editing results. Plotting all efficacy data points for FLAG insertion taken from Figure 4c and Figure 5 shows the correlation between two independently executed experiments (P = 0.0002 for 'PE2', P = 0.0012 for 'Exo-PE'). Gray lines indicate the 0.95 confidence bands of the linear regression. Please see **Supplementary Table 1** for complete statistical results.

FACS gating strategy

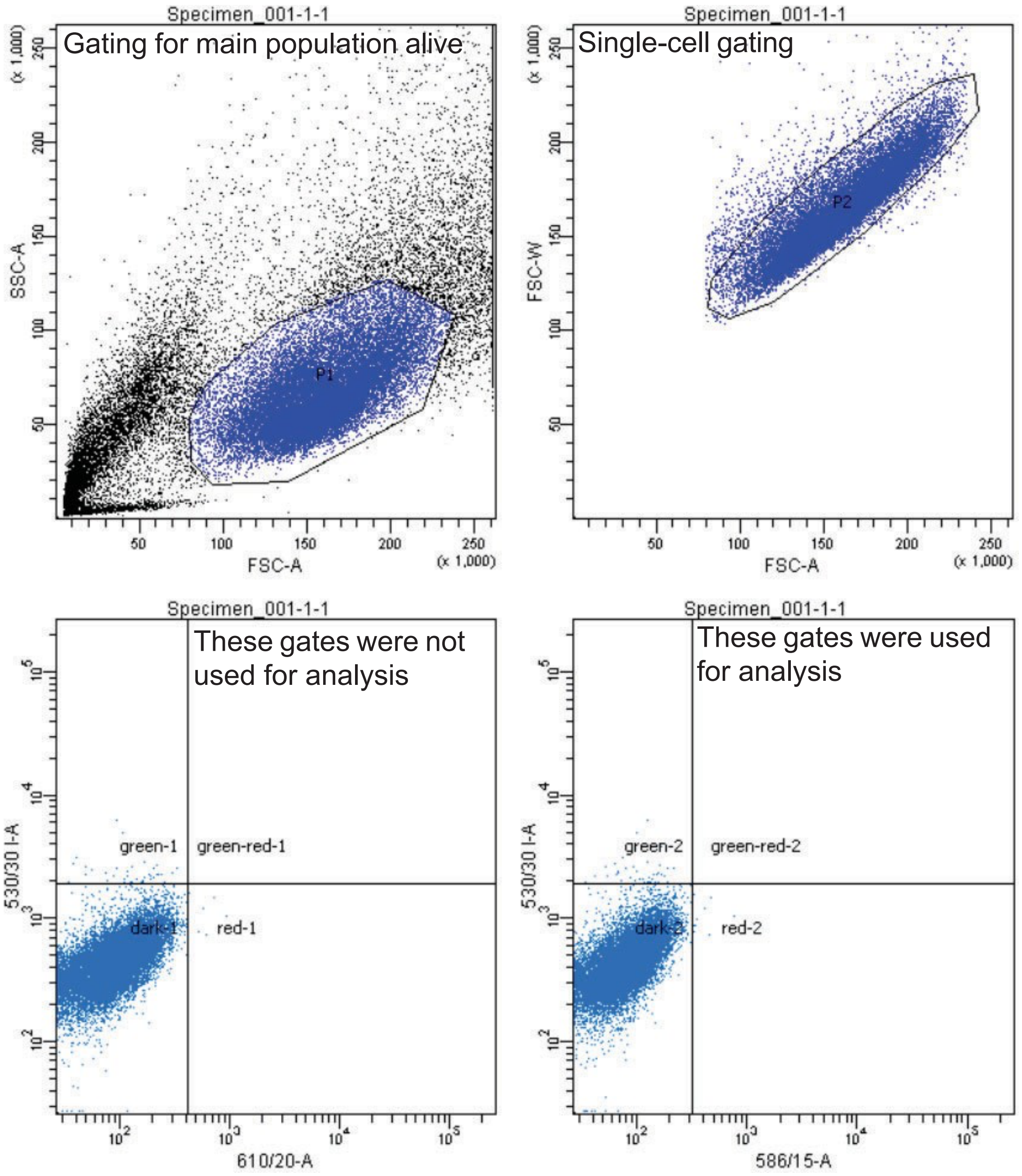

FACS gating strategy

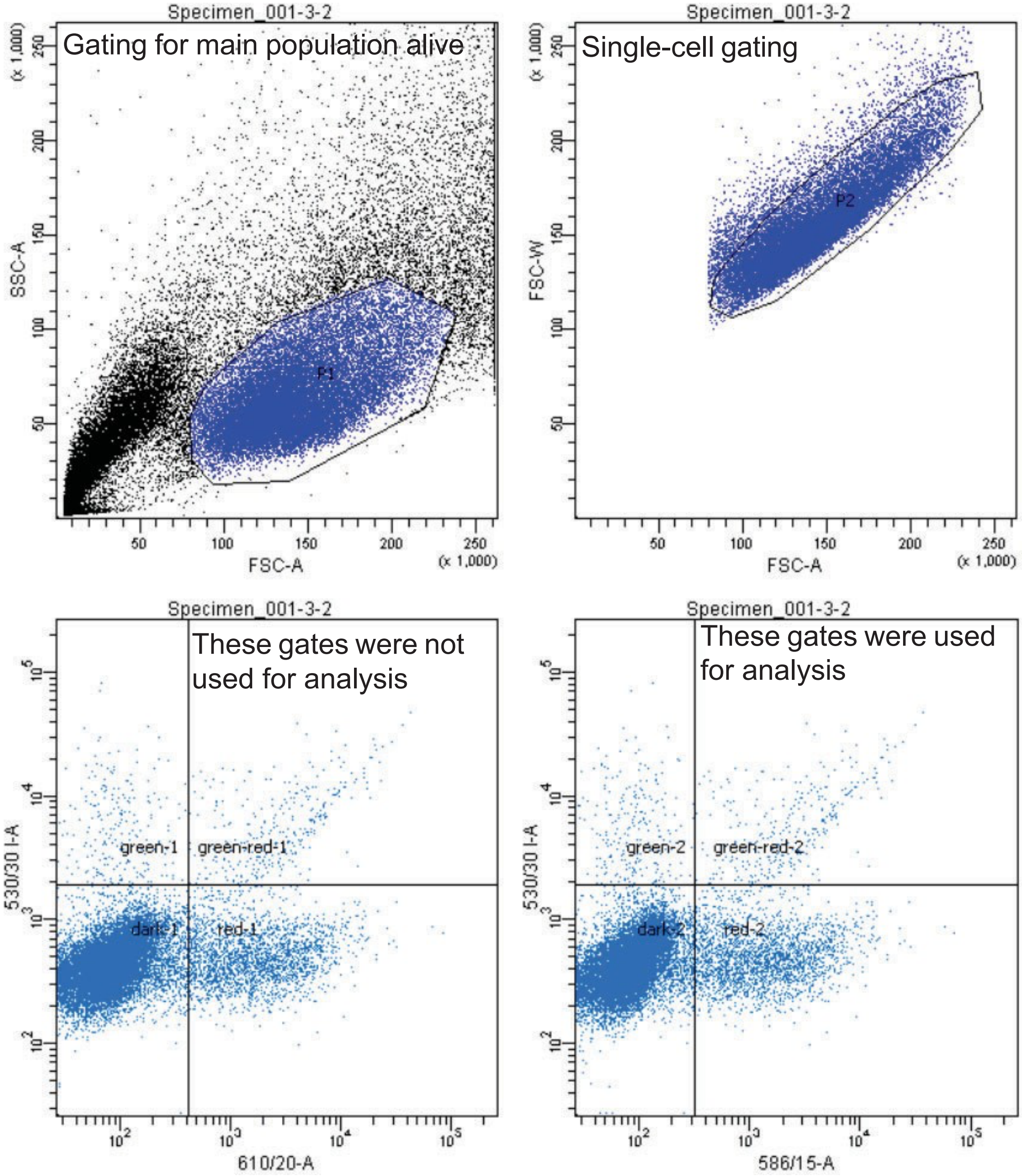

FACS gating strategy

Tube: 3-2

Population	#Events	%Parent	%Total
All Events	50,000	####	100.0
P1	22,672	45.3	45.3
P2	21,245	93.7	42.5
green-1	234	1.1	0.5
green-red-1	299	1.4	0.6
dark-1	18,403	86.6	36.8
[illegible]	2,309	[illegible]	4.6
green-2	239	1.1	0.5
green-red-2	308	1.4	0.6
dark-2	18,278	86.0	36.6
red-2	2,420	11.4	4.8
iRFP720	0	0.0	0.0

Highlighted numbers are used for analysis
