# Supplementary material for: Exonuclease-enhanced prime editors
Source: Nat Methods. 2024 Feb 1;21(3):455–64. doi: 10.1038/s41592-023-02162-w (PMC10927552; doi:10.1038/s41592-023-02162-w)
Supplement: Supplementary file 2 — Reporting Summary [file 41592_2023_2162_MOESM2_ESM.pdf]

nature portfolio

Corresponding author(s): Gil Gregor Westmeyer

Last updated by author(s): Oct 27, 2023

Reporting Summary

Nature Portfolio wishes to improve the reproducibility of the work that we publish. This form provides structure for consistency and transparency in reporting. For further information on Nature Portfolio policies, see our Editorial Policies and the Editorial Policy Checklist.

Statistics

For all statistical analyses, confirm that the following items are present in the figure legend, table legend, main text, or Methods section.

n/a	Confirmed	
☐	☒	The exact sample size (*n*) for each experimental group/condition, given as a discrete number and unit of measurement
☐	☒	A statement on whether measurements were taken from distinct samples or whether the same sample was measured repeatedly
☐	☒	The statistical test(s) used AND whether they are one- or two-sided<br>*Only common tests should be described solely by name; describe more complex techniques in the Methods section.*
☐	☒	A description of all covariates tested
☐	☒	A description of any assumptions or corrections, such as tests of normality and adjustment for multiple comparisons
☐	☒	A full description of the statistical parameters including central tendency (e.g. means) or other basic estimates (e.g. regression coefficient) AND variation (e.g. standard deviation) or associated estimates of uncertainty (e.g. confidence intervals)
☐	☒	For null hypothesis testing, the test statistic (e.g. *F*, *t*, *r*) with confidence intervals, effect sizes, degrees of freedom and *P* value noted<br>*Give P values as exact values whenever suitable.*
☒	☐	For Bayesian analysis, information on the choice of priors and Markov chain Monte Carlo settings
☒	☐	For hierarchical and complex designs, identification of the appropriate level for tests and full reporting of outcomes
☐	☒	Estimates of effect sizes (e.g. Cohen's *d*, Pearson's *r*), indicating how they were calculated

Our web collection on statistics for biologists contains articles on many of the points above.

Software and code

Policy information about availability of computer code

Data collection	Geneious Prime 2023.1.2, BD FACSDiva 6.1.3
Data analysis	Geneious Prime 2023.1.2, BD FACSDiva 6.1.3, NetGene2 (v2.42), R (3.4.1) with emmeans package

For manuscripts utilizing custom algorithms or software that are central to the research but not yet described in published literature, software must be made available to editors and reviewers. We strongly encourage code deposition in a community repository (e.g. GitHub). See the Nature Portfolio guidelines for submitting code & software for further information.

Data

Policy information about availability of data

All manuscripts must include a data availability statement. This statement should provide the following information, where applicable:

- Accession codes, unique identifiers, or web links for publicly available datasets
- A description of any restrictions on data availability
- For clinical datasets or third party data, please ensure that the statement adheres to our policy

Statistical source data are provided with this paper. All other data supporting the findings of this study are available from the corresponding author on request.

Research involving human participants, their data, or biological material

Policy information about studies with human participants or human data. See also policy information about sex, gender (identity/presentation), and sexual orientation and race, ethnicity and racism.

Reporting on sex and gender	n.a.
Reporting on race, ethnicity, or other socially relevant groupings	n.a.
Population characteristics	n.a.
Recruitment	n.a.
Ethics oversight	n.a.

Note that full information on the approval of the study protocol must also be provided in the manuscript.

Field-specific reporting

Please select the one below that is the best fit for your research. If you are not sure, read the appropriate sections before making your selection.

☒ Life sciences ☐ Behavioural & social sciences ☐ Ecological, evolutionary & environmental sciences

For a reference copy of the document with all sections, see nature.com/documents/nr-reporting-summary-flat.pdf

Life sciences study design

All studies must disclose on these points even when the disclosure is negative.

Sample size	Preliminary eTLR-based experiments showed that there was only a small variation over independent replicates, so we decided on n = 3 for subsequent experiments.
Data exclusions	Some NGS data points were excluded because of technical failure (no reads).
Replication	Exo-PE effect were successfully replicated with independent biological samples across multiple loci and cell lines, please see "Statistics and Reproducibility" statement.
Randomization	All of our experiments were performed in multi-well cell culture plates. Wells were allocated to the different treatment groups specified by the particular experiment without determining any specific property of the cells in a given well for selecting a specific condition. Processing of the samples was performed without prior knowledge of what exact condition a given sample belonged to avoid processing bias.
Blinding	All experiments were performed, if possible, with master mixes and with multichannel pipettes to exclude unconscious biases during experimental setup. However, the individual experimenter was not blinded for the conditions as this was not feasible.

Reporting for specific materials, systems and methods

We require information from authors about some types of materials, experimental systems and methods used in many studies. Here, indicate whether each material, system or method listed is relevant to your study. If you are not sure if a list item applies to your research, read the appropriate section before selecting a response.

Materials & experimental systems

n/a	Involved in the study	
☒	☐	Antibodies
☐	☒	Eukaryotic cell lines
☒	☐	Palaeontology and archaeology
☒	☐	Animals and other organisms
☒	☐	Clinical data
☒	☐	Dual use research of concern
☒	☐	Plants

Methods

n/a	Involved in the study	
☒	☐	ChIP-seq
☐	☒	Flow cytometry
☒	☐	MRI-based neuroimaging

Eukaryotic cell lines

Policy information about cell lines and Sex and Gender in Research

Cell line source(s)	HEK293T cells (ECACC: 12022001, Sigma-Aldrich), HEK293 cells (85120602, Sigma-Aldrich), HeLa (93021013, Sigma-Aldrich)
Authentication	All cells were purchased from trusted vendors.
Mycoplasma contamination	Cell lines were regularly tested for mycoplasma contamination with Hoechst 3334, which visualizes non-nuclear cytosolic speckles in case of contamination.
Commonly misidentified lines (See ICLAC register)	No commonly misidentified cell lines were used in this study.

Flow Cytometry

Plots

Confirm that:

☒ The axis labels state the marker and fluorochrome used (e.g. CD4-FITC).

☒ The axis scales are clearly visible. Include numbers along axes only for bottom left plot of group (a 'group' is an analysis of identical markers).

☒ All plots are contour plots with outliers or pseudocolor plots.

☒ A numerical value for number of cells or percentage (with statistics) is provided.

Methodology

Sample preparation	Cells were detached with Accutase, pelleted (200 rcf and 5 min), and resuspended in ice-cold 0.4% formalin for 10 min. Fixed cells were pelleted again (200 rcf and 5 min) and resuspended in ice-cold 200 µl DPBS for FACS analysis.
Instrument	BD FACSaria II (BD Biosciences)
Software	BD FACSDiva Software (Version 6.1.3, BD Biosciences)
Cell population abundance	For FACS analysis, at least 30k events were recorded per conditon.
Gating strategy	The main population of the cells was gated first according to their FSC-A and SSC-A. Secondly, single cells were gated using FSC-A and FSC-W. The final gate (red and green fluorescence) was used to determine the number of undergoing mutEJ or prime editing

☒ Tick this box to confirm that a figure exemplifying the gating strategy is provided in the Supplementary Information.
